# Head and neck nonmelanoma skin cancers: surgical management and debated issues

**DOI:** 10.1097/MOO.0000000000000960

**Published:** 2024-01-03

**Authors:** Vittorio Rampinelli, Aurora Pinacoli, Cesare Piazza

**Affiliations:** Unit of Otorhinolaryngology – Head and Neck Surgery, ASST Spedali Civili, Department of Surgical and Medical Specialties, Radiological Sciences, and Public Health, University of Brescia, School of Medicine, Brescia, Italy

**Keywords:** immunotherapy, nonmelanoma skin cancers, oncologic outcomes, prognosis, surgical margins

## Abstract

**Purpose of review:**

This review critically assesses the current literature and guidelines, aiming to clarify some of the most important factors that impact surgical strategies of head and neck nonmelanoma skin cancers (NMSCs), focusing on squamous, basal, and Merkel cell carcinomas.

**Recent findings:**

Recent developments underscore the complexity of treatment for NMSC, particularly in the head and neck region. There is a lack of high-level evidence for the management of these tumors, especially in advanced stages. The need to tailor the extent of surgical margins and parotid/neck management to different histotypes, considering the varying risk factors for recurrence, is beginning to emerge in the literature. Moreover, the role of immunotherapy and targeted therapies for locally advanced disease, alongsi

de traditional treatment options, is progressively growing.

**Summary:**

NMSCs represent a heterogeneous group of malignancies with varying treatment complexities and prognoses. Management of NMSC is evolving towards an increasingly personalized strategy within a multidisciplinary therapeutic framework.

## INTRODUCTION

Nonmelanoma skin cancers (NMSCs) are the most common malignancies worldwide and the majority of cases primarily affect the head and neck region. They include several pathological entities, the most represented of which are basal (BCC), squamous (SCC), and Merkel cell carcinomas (MCC). Roughly 75–80% of cases consist of BCC, while approximately 20–25% are SCC [1,2]. MCCs are extremely rare, representing less than 5% of the total number of NMSCs. Different histotypes exhibit considerable heterogeneity in clinical behavior and pathologic features. BCC typically has a tendency towards slow growth and limited metastatic potential [3]. On the other hand, SCC usually displays more aggressive behavior with an increased risk of local recurrence, distant spread, and higher rates of mortality. MCC, a rare neuroendocrine skin cancer with a high risk of local recurrence, distant metastasis, and poor prognosis, is even more aggressive [4].

Therapeutic management of NMSCs is planned according to several patient and tumor-related factors, with surgical excision representing the mainstay of treatment regardless of the patient's age and anatomic location of the tumor [5]. The adequacy of surgical margins depends on multiple factors, including histopathological features and tumor size [6]. Anatomic location and individual patient variables such as age, comorbidities, cosmetic expectations, and quality of life should also be considered in the surgical decision-making process. In fact, ablative needs must be balanced with the necessity to guarantee, whenever possible, the functionality and esthetics of the anatomical structures involved [7].

Two additional factors complicate the treatment of patients with head and neck NMSCs. The first is the epidemiologic association of skin tumors to the last decades of life. This is likely to increase the frequency of surgery among elderly patients with several comorbidities [8]. Due to the potentially high rate of postoperative complications in this population, the multidisciplinary group must select the best treatment for each patient, assessing the global status (biological age) and not just chronological age. The second factor is the lack of high-level evidence guiding the management of NMSC, especially in advanced stages. As a consequence, different professionals (i.e., dermatologists, plastic surgeons, otolaryngologists, and maxillofacial surgeons) face this disease with nonuniform indications and competencies. Therefore, it is necessary to integrate the diverse expertise among specialists within a broad multidisciplinary team to work together and share a common clinical reasoning.

This article aims to critically review the existing literature and guidelines to clarify the factors influencing the determination of surgical strategy and highlight key points in treatment of NMSC. Particular focus will be put on SCC, given that this histology is the most typical challenge for the head and neck surgeon, along with frequency and aggressive biologic behavior. Moreover, peculiarities about other cutaneous histotypes will be outlined in dedicated sections, as well as indications for radiotherapy, chemotherapy (CHT), and other emerging types of systemic therapy. 

**Box 1:**
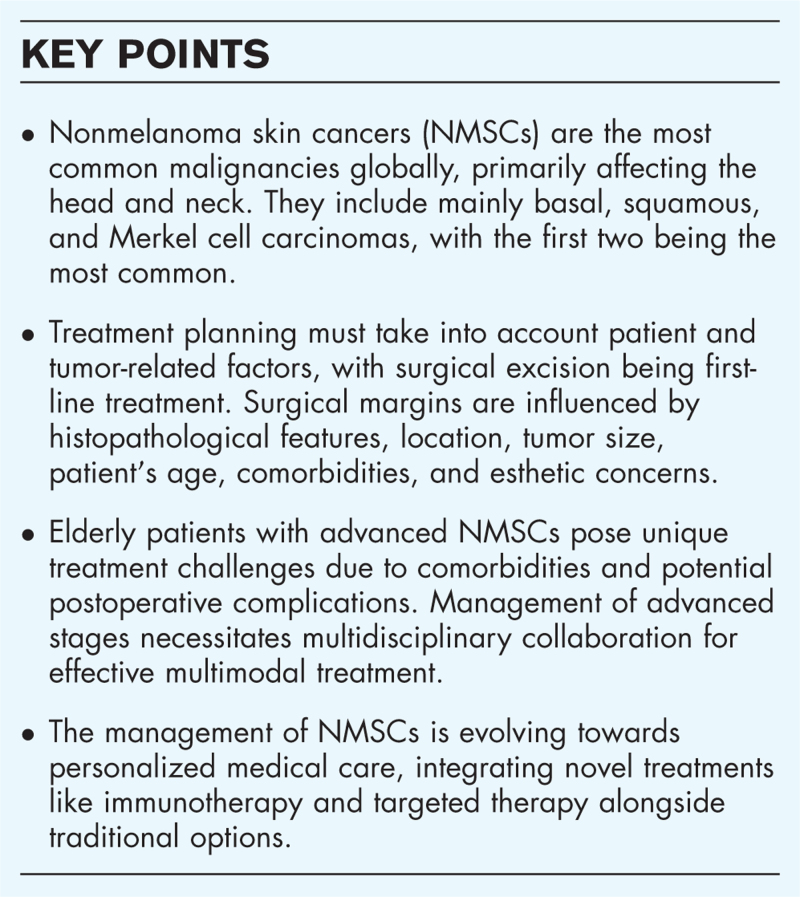
no caption available

## SQUAMOUS CELL CARCINOMA

SCC is a common type of NMSC, frequently affecting the head and neck area, especially in Caucasian patients with a history of life-long exposure to the sun [9]. Based on its extension, cutaneous SCC is divided into common primary and advanced types. The former is a nonmetastatic SCC, which can be further classified as being at low-risk or high-risk for recurrence. Advanced SCCs are further classified as either locally advanced or metastatic [10]. Locally advanced SCCs, at the extreme of the pathologic spectrum, may end up as “nonresectable” tumors due to factors like multiple recurrences and extensive or critical extension to bone and/or major vessels. Moreover, this category includes SCCs whose treatment could lead to unacceptable functional and cosmetic consequences [10]. This concept, however, varies significantly among different specialists involved in the treatment process. Therefore, it is pivotal to discuss advanced cases in a multidisciplinary board, ensuring that tumor resectability, as well as the modality of resection and reconstruction, are agreed upon before considering the patient for nonsurgical or palliative systemic treatments.

The group of metastatic SCCs includes not only cases with distant and in-transit metastases, but also patients with regional lymph node metastases, including those in the parotid. Patients with metastatic SCC should also undergo multidisciplinary evaluation, since regional metastases in selected cases can be successfully treated with unimodal or multimodal therapy, including surgery.

For these reasons, when developing a treatment strategy, the case manager should consider patient's expectations through counseling and critically evaluate the hospital facility and surgical experience of the multidisciplinary group. Centers limited to outpatient surgery under local anesthesia should avoid treating complex cases and refer patients *ab initio* to hubs that are capable of offering comprehensive surgery under general anesthesia and multimodal treatment.

### Risk stratification

The primary goal of any oncologic surgery is to achieve free margins of resection. In NMSCs, the adequacy of margins is influenced by multiple factors, which define the risk of local, regional, or distant recurrence. In recent years, many scores have been developed to stratify these risks for cutaneous head and neck SCCs. The factors considered are both tumor-related (clinical and histological) and patient-related. Moreover, they vary considerably across different authors and experiences as a consequence of the modest scientific evidence supporting risk classification systems [10].

Tumors defined as locally advanced are considered at high-risk by definition, and represent the most critical lesions to be precociously identified to prevent them from being treated suboptimally or evolving into more advanced or even nonresectable tumors [10]. Table 1 gathers the main factors for high-risk cutaneous SCC, divided into clinical and pathological factors [11–16]. The former are elements usually available before surgery and therefore useful in planning the most adequate treatment strategy. Biopsy of a suspicious cancer can provide valuable prognostic information [10]. Therefore, it is beneficial to perform an incisional biopsy on clinically, potentially high-risk lesions. In contrast, an excisional biopsy in the presence of clinical risk factors can result in an oncologically inadequate resection. Widening the margins in patients who have been improperly operated on before, especially if they have undergone complex local reconstructions, is not always feasible and reproducible within well tolerated surgical margins. Clinicians are therefore forced to redirect the patient towards suboptimal radicalization strategies (frequently more aggressive than needed) or risky clinical monitoring.

**Table 1:** Factors characterizing high-risk, head, and neck cutaneous squamous cell carcinomas

	Factor
Clinical factors	Maximum clinical diameter >20 mm^a^^,^^c^ Maximum clinical diameter >40 mm (very high-risk)^a^^,^^b^^,^^c^^,^^d^ [11–14]
	Neurologic symptoms^a^^,^^c^^,^^d^ *Namely pain, numbness, tingling, paralysis, formication, burning, anesthesia, paresthesia, diplopia, and blurred vision as a likely consequence of the impaired function of named nerves passing in the tumor field*. [12,14]
	Primary tumor site *Ear*^a^^,^^b^*, temple*^b^^,^^c^*, lip*^b^^,^^c^ [15]
	Rapidly growing tumor^b^ *Defined as tumor growth at a rate >4 mm/month*. [12,16]
	Poorly defined clinical extent^a^^,^^c^^,^^d^ [12]
	Recurrent tumor^a^^,^^b^^,^^c^ *All recurrent tumors are defined as high-risk irrespective of prior therapy*. [12]
	Immunosuppression^a^^,^^c^^,^^d^ *Especially organ transplant recipients and HIV patients*. [11]
	Bone erosion (very high-risk)^a^^,^^b^^,^^d^ *Intended as radiological evidence of bone involvement*. [11]
Histopathological factors	2–6 mm depth (intermediate/high-risk)^a^^,^^b^ Thickness >6 mm or invasion beyond subcutaneous fat (very high-risk)^a^^,^^b^ [11,12,14,15]
	Positive histological margins (very high-risk)^a^^,^^b^^,^^d^ *One or more involved or close (<1 mm) histological margin (s) in a high-risk tumor*. [11]
	Poor differentiation or desmoplastic (very high-risk)^a^^,^^c^^,^^d^ [11,12,15]
	Perineural invasion^a^^,^^c^^,^^d^ *Tumor cells within the sheath of a nerve deep to the dermis or with a caliber 0.1 mm or larger or clinical and/or radiological involvement of named nerves*. [11–13,15]

a high-risk for local recurrence.

b high-risk for regional recurrence.

c high-risk for distant metastasis.

d high-risk for disease-specific death.

### Surgical margin adequacy

The standard treatment for invasive SCC is surgical removal, ensuring appropriately safe margins of macroscopically normal skin, followed by postoperative histologic examination with scrupulous margins assessment [5,17^▪▪^]. Intraoperative frozen sections are useful to ensure clear resection margins, especially in high-risk cases. However, the possibility of false negatives should be always considered, and definitive postoperative analyses performed with paraffin-embedded sections [5]. Moreover, in cases with a high probability of incomplete resection, when feasible, delayed reconstruction should be preferred [5,18].

Alongside conventional surgery with safe margins, micrographically controlled surgeries, such as Mohs surgery and 3D histology, can be employed in high-risk cutaneous head and neck SCCs. These techniques, which involve intraoperative frozen sections and paraffin-embedded section analysis, respectively, have shown good results in terms of surgical radicality and preservation of healthy tissue [5]. However, they are time-consuming, expensive, and less suited for large lesions necessitating nondelayed reconstruction. For these reasons, conventional surgery is most widely used.

Adequacy of margins is influenced by the presence of the above-mentioned risk factors for recurrence. Low-risk common primaries benefit from 4 to 6 mm clear margins. Clinically well defined cutaneous SCCs with diameters under 2 cm have been shown to be successfully treated in approximately 95% of cases by resection within 4 mm margins [5,19,20]. For high-risk SCCs, wider margins are recommended, varying between 6 and 13 mm according to different guidelines [5,21–24]. The European consensus group suggests safe margins range from 6 to 10 mm, while the latest 2024 National Comprehensive Cancer Network (NCCN) guidelines recommend determining margin adequacy on a case-by-case basis, factoring in tumor and patient-specific features [12,17^▪▪^,^23^]. This approach is particularly relevant in the head and neck area, where achieving wide resection margins without compromising important structures and functions can be challenging. Additionally, the concentration of vascular, nervous, muscular, and bone structures in this area provides the tumor with preferential escape routes, which must be carefully considered when planning surgical excision.

The deep margin of resection is usually the most critical to manage, while achieving a wide superficial excision is generally feasible. Key neurovascular structures (especially branches of the facial nerve) lying immediately beneath subcutaneous tissue may limit deep surgical aggressiveness to avoid significant postoperative sequelae. Dealing with deep structures usually requires general anesthesia, advanced technologies (e.g., intraoperative neuromonitoring), and a high level of expertise. Additionally, advanced surgery often involves complex reconstructions with local or free flaps, especially when full-thickness resection of bone and cartilage is performed. If periosteum, perichondrium, bone, and cartilage are removed, simple skin grafts are usually not feasible, leading many surgeons to prefer an inadequate resection in favor of an easier reconstruction.

Each tumor and patient, as well as expectations, must be individually and critically analyzed. Moreover, each subsite of the head and neck area shows peculiarities regarding resection and reconstruction. The scalp presents its own set of complex management challenges. In the presence of radiological evidence of bone infiltration, partial or complete removal of the cranial bone may be necessary, with consequent complex reconstruction. Bone marrow calvarian involvement calls for complete bone resection with dura exposure. Depending on the defect size, a rigid bone reconstruction may be indicated. For limited involvement of the external cortex, outer table calvarian resection offers a well tolerated approach with reasonable oncologic control [25,26]. If the pericranium is clearly involved, its resection in continuity with the outer table of the calvaria should be considered [27]. In high-risk SCCs without evidence of pericranial involvement, a resection without its removal can hardly be considered oncologically adequate. In such scenarios, in fact, resection of the pericranium contiguous to the specimen is strongly indicated to ensure a deep safe margin of clearance. Final histopathologic examination provides key data about margins of resection, allowing the multidisciplinary team to decide whether to continue the treatment process with adjuvant treatment.

The nose, being a central and aesthetically significant facial structure, is affected in up to 25% of NMSC cases of the head and neck region [28]. The treatment of choice for early-stage cancers is simple surgical excision, while advanced stages can benefit from a multidisciplinary evaluation and a multimodal approach. Cutaneous SCCs of the external nasal pyramid differ in biological behavior, treatment strategy, and prognosis from tumors of the same histology originating from the nasal vestibule. Therefore, careful evaluation of the site of tumor origin is necessary, although this can be challenging in advanced cases. Most advanced lesions requiring partial or total rhinectomy are at high-risk of recurrence, especially when previously resected inadequately. This may result from a failure to remove cartilage and reluctance to create a full-thickness defect [29]. The literature suggests considering delayed reconstruction in such high-risk cases [30].

SCCs account for 5–10% of eyelid tumors, which is also a typical location for BCC. To date, the standard of care for periocular malignancies is Mohs micrographic surgery, which allows sparing precious eyelid tissue while ensuring adequate tumor ablation. Orbital invasion by NMSC is considered rare [31], but, when present, results in significant morbidity and mortality [32]. It is usually associated with recurrent disease or incompletely excised medial canthal tumors. Two main pathways for orbital invasion are perineural spread and direct extension [33]. Orbital exenteration, which involves the complete excision of periorbital tissues and orbital contents [34], is indicated for tumors showing invasion of the orbital apex, retrobulbar fat, extraocular muscles, conjunctiva, or sclera [35].

Cutaneous SCCs of the external auditory canal with temporal bone involvement present a challenge for the multidisciplinary team. These aggressive tumors, usually associated with poor prognosis, require multimodal treatment strategies and adequate otoneurosurgical expertise. Due to the complexity of this topic, it will not be further explored herein.

### Neck management

SCCs exhibit the tendency to develop regional lymph node metastases, which typically become clinically evident after treatment of the primary lesion, rather than at its first presentation and evaluation [36,37]. The reported rate of regional metastases in the head and neck varies between 2.3 and 5.2%, depending on the duration of follow-up [38]. These seemingly low rates must be counterbalanced by the absolute number of these tumors in a steadily aging population. Additionally, the development of lymph node metastasis is a prognostically adverse factor, significantly influencing the rate of mortality [37,39]. This has recently led the scientific community to focus on this topic. However, given the low quality of data, generally collected in retrospective studies, there is limited consensus on the ideal management of the neck. Furthermore, treatment guidelines typically provide general indications for all sites, without focusing on the peculiarities of the head and neck anatomical area.

A number of clinicopathological factors have been recognized as potential indicators of an increased risk for metastasis [37], as reported in Table 2[36,40–44]. The patterns of spread can vary greatly between individuals. The facial drainage pathways differ from those typical of mucosal tumors, and the superficial drainage, mainly following the perifacial, periparotid, and external jugular chains, is often unpredictable. Moreover, patients who have undergone multiple skin incisions, by definition present altered pathways of lymphatic drainage. However, approximate predictions about the location of nodes metastasis can be made. The lateral aspects of the head and forehead primarily metastasize to the parotid and level II. Lesions on the neck skin drain preferentially to level II and external jugular nodes. Cancers of the posterior aspect of the head usually spread to occipital nodes and level IIB and V and, occasionally, to the posterior part of the parotid gland. The antero-inferior aspect of the face drains mainly to the submental and submandibular (levels IA and IB) and all the other supra-omohyoid lymph nodes [45].

**Table 2:** Risk factors for lymph node metastasis

	Risk factor
Clinical factors	Immunosuppression [15,36]
	Primary tumor recurrence [40]
	Lesion location [15,36,41,42]: -eyelid -lip -ear * -*cheek -temple
	Age 80–89 years and ≥ 90 years [36]
	Male sex [36]
Histopathologic factors	DOI >6 mm or beyond the subcutaneous fat [15]
	Noncohesive invasive front [43]
	LVI [43]
	Tumor budding [44] *Defined as the presence of either isolated single cells or small cell clusters scattered in the stroma ahead of the invasive tumor front*.
	Poor and moderate differentiations [15]
	Incomplete excision margins [32,44]
	Tumor size [40]
	Tumor volume [43]
	Cartilage invasion [43]
	PNI [40]
	Desmoplasia [44]

DOI, depth of infiltration; LVI, lympho-vascular infiltration; PNI, perineural infiltration.

Vauterin *et al.*[45] analyzed the patterns of lymph node spread of cutaneous SCC in a cohort of 209 patients with clinical evidence of regional metastatic disease. Eighty-two percent of patients showed parotid involvement, 18% neck involvement, and 13.4% clinical evidence of disease in both areas. Among pathologically positive specimens, level II was the most frequently involved (79%). Level IV (13%) and level V (17%) were only involved in extensive lymph nodes disease. Isolated level V metastases exclusively originated from lesions of the posterior scalp [45,46].

Regarding surgical management, in cases of clinically positive parotid and neck involvement, complete parotidectomy and comprehensive neck dissection are indicated. In cases with clinically evident parotid involvement but a negative neck, supra-omohyoid neck dissection, including the external jugular drainage pathway, is considered appropriate. In cases with a posterior primary and any clinically positive node, level V and occipital nodes should be included in the dissection, even if not macroscopically involved [45,47].

The consensus on elective neck dissection in cutaneous head and neck high-risk SCC with a clinically negative neck is limited due to several factors. The rate of occult metastases and their distribution in lymph nodes levels (including the parotid) varies greatly in the literature, with reported rates ranging from 0 to 37%, and an average between 20 and 30% [37,46,48,49]. These percentages are influenced by the aforementioned risk factors, but there are no reliable models that are capable of predicting the probability and possible localization of occult metastasis to plan an elective dissection that would be beneficial for survival. Prophylactic neck clearance has been demonstrated to offer better survival outcomes compared to elective nodal irradiation when the probability of occult metastasis is 30% or higher [50]. Nevertheless, the relative level of evidence is low, and the risks of metastasis are difficult to precisely quantify. However, personalized patient assessment considering risk factors for metastasis and location of the primary tumor allows for selection of cases where prophylactic neck dissection, with or without parotidectomy, may be advantageous [10,51]. Additional elements favoring prophylactic dissection include the tumor's contiguity with the parotid gland (direct infiltration or proximity), suggesting the need for parotidectomy to achieve a deep free margin. Similarly, if identifying the peripheral branches of the facial nerve is necessary to remove the primary tumor, parotidectomy would serve the dual purpose of exposing the nerve trunks and removing potentially involved lymph nodes. Moreover, if a free flap reconstruction is planned, prophylactic neck dissection would occur in the context of exposing the recipient vessels for microvascular anastomoses [47].

Regarding the extent of prophylactic dissection, the data in the literature are limited, also due to the inter-individual variability of lymphatic drainage. However, the dissection can be planned by considering the rate and distribution of regional metastases, typically at the parotid and level II, followed by levels I and III. Level V should be considered in cases of primary lesions with a posterior location [37,52].

## BASAL CELL CARCINOMA

BCC is a slowly growing, locally invasive skin cancer that commonly affects individuals with a significant history of sun exposure or Gorlin syndrome [53]. Surgical excision is the most prevalent and effective treatment for BCC. Mohs micrographic surgery and radiotherapy may be indicated in selected cases, such as when there is a need to spare healthy tissue in critical areas (e.g., the eyelid) or when surgical treatment has been refused [54].

### Risk stratification

Several factors traditionally associated with BCC aggressiveness influence the risk of recurrence and prognosis. These include a tumor size greater than 2 cm, usually associated with extensive subclinical invasion of adjacent tissues [55]; location in high-risk areas such as the nose and peri-orificial areas of the face, while intermediate-risk zones include the forehead, cheeks, chin, scalp, and neck; lesions with poorly defined margins clinically; histological types such as morpheiform, infiltrating, and metatypical variants; histological features like perineural and lympho-vascular invasion; deep infiltration (reticular dermis and subcutis); relapse after previous treatment; and immunosuppression [54]. The therapeutic strategy as well as the adequacy of surgical margins are tailored based on these risk factors. Other considerations in the decision-making process include the patient's age, comorbidities, and number of lesions present. The recently updated European Dermatology Forum (EDF) and European Association of Dermato-Oncology (EADO) guidelines propose a simplified and operational classification of BCCs into ’easy to treat’ and ’difficult to treat’ categories. Factors that make BCCs difficult to treat include technical issues in maintaining function and esthetics, ill-defined boundaries, multiple recurrences, or prior radiotherapy [53].

### Surgical margin adequacy

The NCCN guidelines recommend a resection with a 4 mm margin for well defined, low-risk tumors [56], while the European EDF/EADO guidelines suggest a range of peripheral margins between 2 and 5 mm [53]. A meta-analysis including 16 066 lesions demonstrated that a 3 mm surgical margin is safe in achieving a 95% cure rate for nonmorpheiform lesions 2 cm or less [56]. High-risk lesions require wider margins of resection, between 4 and 6 mm and 5 and 15 mm for the NCCN and EDF/EADO guidelines, respectively [53,56].

There is a lack of consensus about the management of the deep resection margin. Guidelines [53,57] recommend an excision down to the level of the fascia, perichondrium, or periosteum, without further specifications. It is the authors’ opinion that the principles of deep margin management for SCCs are also appropriate for BCCs. Recurrence after surgery of incompletely excised BCCs ranges, in fact, from 26 to 41% after 2–5 years of follow-up [58].

## MERKEL CELL CARCINOMA

MCC is an aggressive neuroendocrine skin cancer, which usually arises in sun-damaged regions. It has been linked to immunosuppression and infection with Merkel cell polyomavirus [59]. Head and neck MCC is characterized by high rates of nodal involvement, local recurrence, and distant metastasis, with size greater than 1 cm, immunosuppression, and primary head and neck site considered as risk factors associated with scarce prognosis [60].

Wide local excision with individualized margins based on local extension and multimodal therapy is usually the treatment of choice [60]. Surgery has been shown to produce superior outcomes compared to nonsurgical primary treatment [60]. According to the guidelines from the NCCN and EDF/EADO, resection margins of 1–2 cm are recommended [60,61]. No precise statement on the ideal width of the resection margins between 1 and 2 cm is made to date because studies have shown no differences in the recurrence-free interval [61,62].

Interestingly, margin status affects survival and recurrence predominantly for Stage I and II tumors, while it is not relevant for more advanced lesions [60]. However, given the rarity of such a histology, it must be underlined that there is a lack of substantial evidence in the literature, and no prospective trials have been conducted to assess the variations in recurrence and survival rates based on specific resection margin widths.

## RADIOTHERAPY

Although the standard of care for head and neck NMSC is surgical resection, radiotherapy plays a significant role in both definitive and adjuvant settings.

For cutaneous SCCs not suitable for surgery due to invasion of vital structures or patient-related factors, definitive radiotherapy should be considered based on multidisciplinary evaluation [17^▪▪^]. The level of evidence supporting the efficacy of radiotherapy in the adjuvant setting is limited, with mostly retrospective data available. However, in advanced cases, it often becomes the sole alternative, whether used in combination with systemic therapies including immunotherapy, CHT, or targeted therapies, or not [17^▪▪^]. Adjuvant radiotherapy can represent an alternative in cases with positive tumor margins at definitive histologic examination. Although suboptimal compared to re-excision, this option can be accepted in presence of complex reconstructions or compromised patients [17^▪▪^]. In case of high-risk R0 cases, the benefit in terms of survival of postoperative radiotherapy is debated [17^▪▪^,^63^,^64^]. In addition to margin status, perineural invasion at definitive histologic examination leads to adjuvant treatment in many centers [17^▪▪^]. Moreover, adjuvant radiotherapy following neck dissection should be considered in SCC with multiple regional nodal metastases and extracapsular extension [17^▪▪^].

Patients with BCCs benefit from adjuvant radiotherapy in case of persistence of positive margins after multiple resections not amenable of re-excision, clinically evident perineural involvement, or in case of T4 tumors with extensive bone and soft tissues invasion [53,65].

Radiotherapy is a feasible alternative to surgical intervention, especially for elderly patients who are unsuitable for surgery due to underlying health conditions or refusal of surgery [53]. In these settings, radiotherapy has shown good rates of cure, comparable to those of surgery in terms of local control [66]. Moreover, radiotherapy is considered in the palliative setting to improve the patient's quality of life when advanced lesions are not amenable for curative treatment [53].

MCC is highly sensitive to radiotherapy [61]. Postoperative irradiation has shown good prognostic results in all MCC except for R0, N0, Stage I lesions without lympho-vascular invasion [61,67]. Moreover, prophylactic radiotherapy to the regional nodes has been associated with increased regional relapse-free survival, although without impact on overall survival [61,68].

## SYSTEMIC THERAPY

Patients with advanced disease generally have poor prognosis and benefit from evaluation and management by a multidisciplinary tumor board. Even with limited clinical experience, the role of immunotherapy and targeted therapy as promising treatments for locally advanced NMSCs is gaining prominence [69,70]. For patients with locally advanced or metastatic SCCs who are not candidates for curative local treatment, first-line therapy with a PD-1 agent, such as cemiplimab, should be considered [17^▪▪^]. Additionally, epidermal growth factor receptor inhibitors, like cetuximab, are being used as secondary systemic treatments for advanced SCC, often combined with CHT or radiotherapy [17^▪▪^].

Systemic therapy may be beneficial for difficult-to-treat BCCs. For patients with unresectable and metastatic BCC, Hedgehog inhibitors, such as vismodegib or sonidegib, are recommended. In cases of disease progression or contraindication to these inhibitors, immunotherapy with cemiplimab is considered as second-line treatment [53]. PD-1/PD-L1 inhibitors have shown good responses also in patients with advanced MCC [60].

Although the introduction of immunotherapy and targeted therapy is transforming the treatment landscape for patients with advanced NMSC, several crucial aspects require further research [71^▪^]. One of the most intriguing questions is how these treatments can be integrated into a curative framework approach. The NCCN guidelines suggest considering neoadjuvant cemiplimab for SCC with nodal metastasis and unresectable or resectable with high morbidity [17^▪▪^]. Similarly, cemiplimab and nivolumab have shown promising results in a neoadjuvant setting for advanced BCCs [7].

Apart from the clinical responses to treatment, which vary based on literature reports [71^▪^], the benefits of integrating surgery and RT after neoadjuvant treatment remains unclear. To date, there are no supporting data for a resection limited to the extent of postinduction disease compared to one encompassing the original disease boundaries. However, with the increasing use of these drugs, it is expected that these fundamental aspects of treating advanced tumors within a multimodal therapeutic framework will become clearer in the coming years.

## CONCLUSION

NMSCs represent a group of malignancies with varying treatment complexities and prognoses. The management of these cancers, especially in the head and neck region, requires a multidisciplinary approach and consideration of patient- and tumor-specific factors. Surgical excision remains the cornerstone of treatment, with the goal of achieving clear margins, although the extent of resection is guided by the characteristics and location of the tumor.

The evolution in the management of NMSCs is particularly evident in the rising role of immunotherapy and targeted therapy for advanced, unresectable cases. However, the integration of these newer modalities with conventional surgery and radiotherapy remains an area of ongoing research and discussion. This comprehensive review underscores the importance of individualized care, the need for continued research, and the value of a multidisciplinary approach in managing the complex spectrum of NMSCs of the head and neck.

## Acknowledgements

*None.*

### Financial support and sponsorship

*None.*

### Conflicts of interest

*There are no conflicts of interest.*

## REFERENCES AND RECOMMENDED READING

Papers of particular interest, published within the annual period of review, have been highlighted as:

▪ of special interest
▪▪ of outstanding interest
